# Multifactorial analysis of the stochastic epigenetic variability in cord blood confirmed an impact of common behavioral and environmental factors but not of in vitro conception

**DOI:** 10.1186/s13148-018-0510-3

**Published:** 2018-06-08

**Authors:** D. Gentilini, E. Somigliana, L. Pagliardini, E. Rabellotti, P. Garagnani, L. Bernardinelli, E. Papaleo, M. Candiani, A. M. Di Blasio, P. Viganò

**Affiliations:** 1 Istituto Auxologico Italiano IRCCS, 20095 Cusano Milanino, Italy; 2 Infertility Unit, Fondazione Ca’ Granda, Ospedale Maggiore Policlinico, 20122 Milan, Italy; 3 Reproductive Sciences Laboratory, Division of Genetics and Cell Biology, IRCCS Ospedale San Raffaele, Via Olgettina 58, 20132 Milan, Italy; 4 Department of Experimental, Diagnostic and Specialty Medicine, University of Bologna, 40138 Bologna, Italy; 5 Department of Brain and Behavioral Sciences, University of Pavia, 27100 Pavia, Italy; 6 Obstetrics and Gynaecology Unit, IRCCS Ospedale San Raffaele, 20132 Milan, Italy

**Keywords:** Epigenome-wide analysis, Assisted reproduction technology, DNA methylation, Imprinted genes, Multiple factor analysis, Stochastic epigenetic variations

## Abstract

**Background:**

An increased incidence of imprint-associated disorders has been reported in babies born from assisted reproductive technology (ART). However, previous studies supporting an association between ART and an altered DNA methylation status of the conceived babies have been often conducted on a limited number of methylation sites and without correction for critical potential confounders. Moreover, all the previous studies focused on the identification of methylation changes shared among subjects while an evaluation of stochastic differences has never been conducted. This study aims to evaluate the effect of ART and other common behavioral or environmental factors associated with pregnancy on stochastic epigenetic variability using a multivariate approach.

**Results:**

DNA methylation levels of cord blood from 23 in vitro and 41 naturally conceived children were analyzed using the Infinium HumanMethylation450 BeadChips. After multiple testing correction, no statistically significant difference emerged in the number of cord blood stochastic epigenetic variations or in the methylation levels between in vitro- and in vivo-conceived babies. Conversely, four multiple factor analysis dimensions summarizing common phenotypic, behavioral, or environmental factors (cord blood cell composition, pre or post conception supplementation of folates, birth percentiles, gestational age, cesarean section, pre-gestational mother’s weight, parents’ BMI and obesity status, presence of adverse pregnancy outcomes, mother’s smoking status, and season of birth) were significantly associated with stochastic epigenetic variability. The stochastic epigenetic variation analysis allowed the identification of a rare imprinting defect in the locus GNAS in one of the babies belonging to the control population, which would not have emerged using a classical case-control association analysis.

**Conclusions:**

We confirmed the effect of several common behavioral or environmental factors on the epigenome of newborns and described for the first time an epigenetic effect related to season of birth. Children born after ART did not appear to have an increased risk of genome-wide changes in DNA methylation either at specific loci or randomly scattered throughout the genome. The inability to identify differences between cases and controls suggests that the number of stochastic epigenetic variations potentially induced by ART was not greater than that naturally produced in response to maternal behavior or other common environmental factors.

**Electronic supplementary material:**

The online version of this article (10.1186/s13148-018-0510-3) contains supplementary material, which is available to authorized users.

## Background

Currently, there is still poor consensus on the possibility that assisted reproduction technology (ART) could affect the epigenome of in vitro-conceived babies [1–3].

Most of the studies addressing the DNA methylation status of children conceived through in vitro fertilization (IVF) with or without intracytoplasmic sperm injection (ICSI) have specifically evaluated alterations in imprinted regions [1, 2]. This on the basis of a suggested link between ART and imprinting disorders. A recent meta-analysis including the results of all the studies regardless of the type of the imprinting disorder, showed a significant association between imprinting diseases and ART (odds ratio = 3.67; 95% confidence interval = 1.39–9.74) [2]. Beckwith-Wiedemann syndrome, as an example, has an estimated worldwide frequency of 1 in 13,700 naturally conceived babies and a weighted relative risk of 5.2 in children conceived by ART [2, 4]. Therefore, there is evidence to suggest an adverse effect of ART procedures, as well as of the underlying subfertility, on imprinting status. The biological rationale behind this idea is that because of the dynamic epigenetic reprogramming occurring during oocyte growth and preimplantation development, environmental perturbations during this time period may affect imprinting establishment and maintenance. Proper allelic expression of imprinted genes are known to play an important role in embryonic and neonatal growth, placental function, and postnatal behavior [1, 2, 4–7].

On the other hand, no evidence of generalized changes in DNA methylation of the imprinted genes *KvDMR/KCNQIOTI*, *PEGI/MESR*, *IGF2*, *PEG3*, and *H19* in association with ART was found [2]. For instance, results of a meta-analysis of four studies comparing percentage methylation of *IGF2* locus between ART and spontaneously conceived babies did not show any significant difference [2]. Although imprinting syndromes are usually associated with profound methylation changes, even the smaller scale changes that could be caused by ART methodologies could potentially predispose to epigenetic alterations at the key loci associated with the syndromes. However, it is unlikely that these hypothetical changes in the methylation status of newborns will be located in genomic regions shared among subjects. Considering that the reprogramming mechanism is driven by a relatively small number of “players” [8], it is difficult to figure out a mechanism where a potential interference related to IVF techniques will result in a small localized damage instead of a stochastic and more widespread effect. Indeed, the stochastic nature of these events has been previously suggested based on data both in humans and animal models [9, 10]. In addition to a potential damage, a stochastic effect could also be related to a rescue mechanism similar to the one observed in the germinal cells of the progeny of Dnmt3L2/2 female mice, where a hierarchy of factors involved in the establishment and maintenance of maternal germline imprints has been found, the loss of one possibly rescued in a stochastic fashion by the activity of the others [11].

Given the supposed small and stochastic effects associated with ART, the analysis of mean methylation levels may not be ideal in exploring differences between in vivo- and in vitro-conceived children. This analysis may be useful in identifying epigenetic alterations shared by a group of subjects and potentially associated with their phenotype but would not reflect differences in individual variation or other features of the methylation spectrum. Rare stochastic epigenetic variations that are not shared among subjects fail to be identified by a classic Epigenome Wide Association Study (EWAS) based on mean methylation values comparison [12]. To overcome this problem, we have conducted an analysis of stochasticity based on the previously introduced concept of stochastic epigenetic variations (SEVs) [12]. SEV is defined as a single CpG with a methylation level detected as an outlier when compared to the methylation level found for the same CpG in a control population. This allows to obtain both a measure of stochasticity at a whole-genome level (total number of SEVs) and a topographic information on genomic regions with an enriched number of SEVs. A previous application of this method permitted to obtain an estimation of the number of epigenetic alterations produced by aging, revealing an exponential association between age and number of SEVs [12]. Only four studies have previously evaluated DNA methylation at a genome-wide level in cord blood of human offspring from ART procedures, and this was done based on the comparison of the mean methylation levels [10, 13–15]. Importantly, correction for phenotypic parental or fetal traits was limited to none or very few confounders although parental BMI, mode of delivery, birth weight, smoke, and intrauterine growth restriction have been previously linked to changes in methylation levels of several genes [16–19].

In this report, we present different analytical approaches in evaluating global DNA methylation of umbilical cord blood from in vitro and naturally conceived newborns mostly with the aim to measure random epigenetic variations and the impact of potential confounders on their number.

## Results

### Dimensionality reduction of all phenotypic traits using multiple factor analysis

**Fig. 1:**
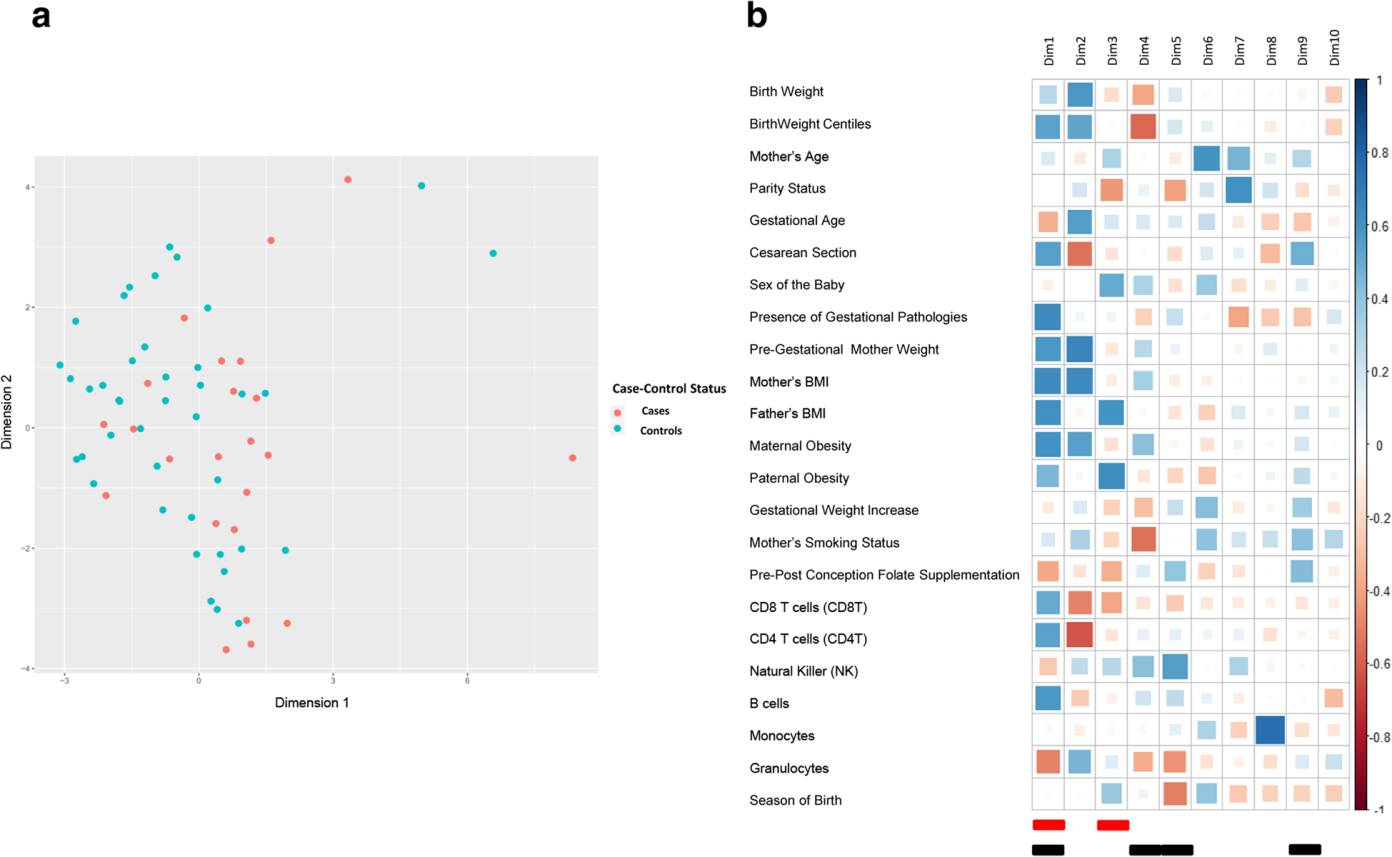
Dimension reduction of phenotypic, behavioral and environmental traits. Dimension reduction obtained by multiple factor analysis of mixed data is used to resume the complexity of all phenotypic, behavioral, and environmental traits. The scatter plot in (**a**) visualizes the samples into a two-dimensional space using the first two dimensions. **b** shows a correlogram indicating correlation levels between dimensions and all the phenotypes used in the multiple factor analysis. The highest correlation indicates that those traits are captured by that specific dimension. Dimensions highlighted in red were significantly associated with case-control status while dimensions highlighted in black were significantly associated with the number of SEVs

Differences at phenotypic level between naturally conceived (*n* = 41) and in vitro-conceived babies (*n* = 23) were evaluated. The phenotypic traits considered were birth weight, birthweight centiles, mother’ s age, parity status, gestational age, cesarean section, sex of the baby, presence of adverse pregnancy outcomes (diabetes, preeclampsia and placenta praevia), pre-gestational mother weight, parents’ BMI and obesity, gestational weight increase, maternal smoking status, pre-post conception folate supplementation, blood cell composition [CD8 T Cells (CD8T), CD4 T Cells (CD4T), natural killer (NK) cells, B cells, monocytes, granulocytes], and season of birth. The correlogram in Additional file 1: Figure S1 shows all the phenotypes analyzed and the presence of a consistent degree of correlation among traits. The number of dimensions to use in the analysis was reduced using the multiple factor analysis of mixed data. Considering the first two obtained dimensions (Dims), the overlap of cases and controls shown in Fig. 1a suggested a phenotypic similarity between the two groups. Taken together, the first 10 dimensions collected 72% of the total variability among the subjects analyzed. Correlations between dimensions and phenotypic traits were evaluated and are shown in Fig. 1b. The logistic regression analysis indicated that the Dim 1 and Dim 3 were significantly associated with case-control status (*p* = 0.03 and *p* = 0.01, respectively) and were subsequently used as covariates in the case-control differential methylation analysis. Phenotypic traits that were mainly captured by Dim1 and Dim 3 were highlighted by elevated correlation levels (Fig. 1b).

### Visual inspection of DNA methylation level using principal component analysis (PCA)

**Fig. 2:**
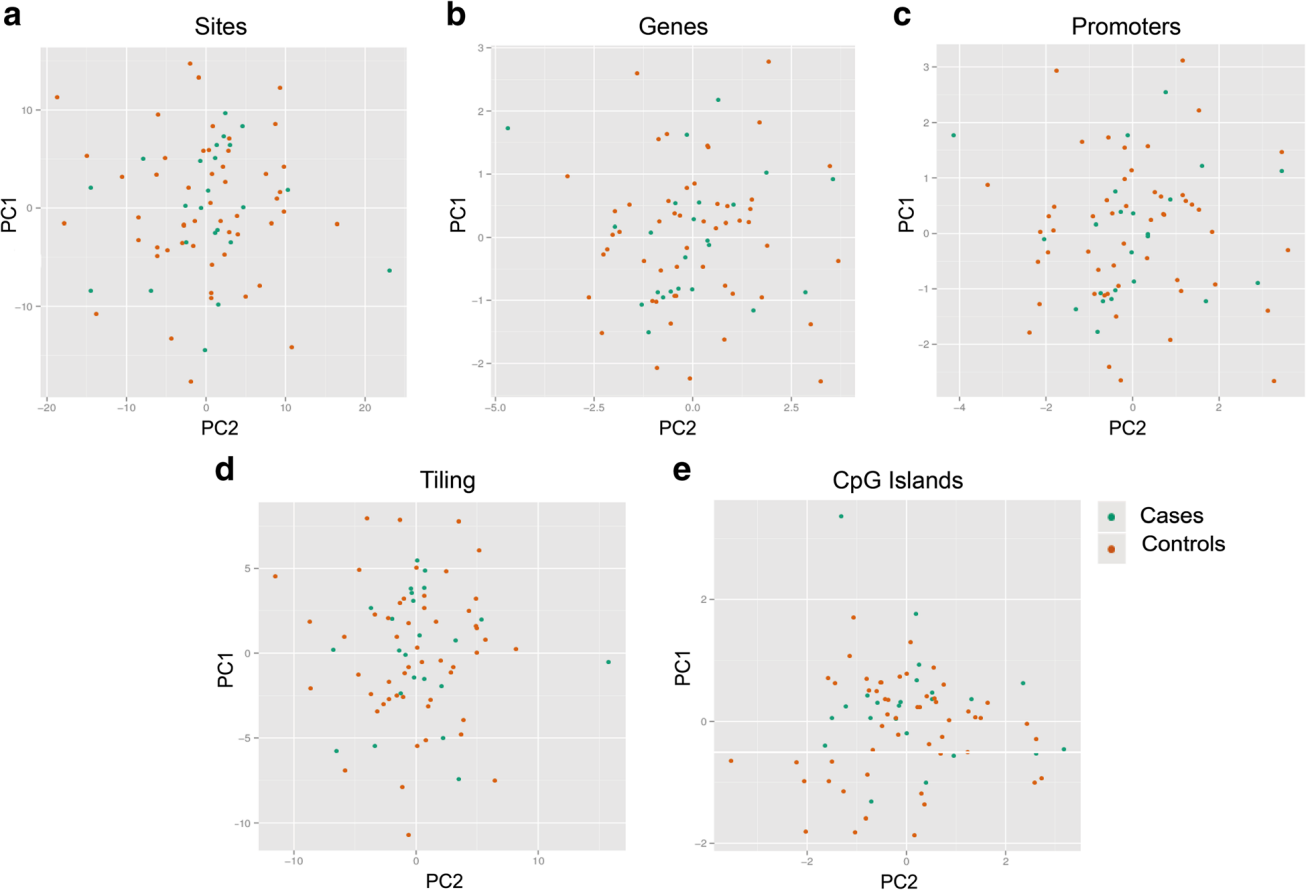
Dimension reduction of methylation data. Dimension reduction is used to visually inspect the dataset for a strong signal in the methylation values that is related to sample clinical or chip batch effects. Values of the first two principal components (PC) in scatter plots are shown considering methylation levels of CpG sites (**a**) and methylation levels of regions such as genes (**b**), promoters (**c**), tiling (**d**), and CpG islands (**e**)

The genome-wide DNA methylation analysis of cord blood was performed using the Infinium HumanMethylation450 BeadChip. Dimensional reduction was used to visually inspect the dataset for strong signals in the methylation values. The PCA was performed considering methylation signals from single CpG sites and also considering four sets of genomic regions: genes, promoters, CpG islands, and tiling (not overlapping regions of 5 kb length). Results reported in Fig. 2 show that there was no strong difference in the methylation level between naturally conceived and in vitro-conceived babies considering both sites and genomic regions.

### Differential methylation analysis

Differential methylation analysis was computed both for site (single CpG) and region level. Dimensions obtained from the multifactorial analysis, which were significantly associated with the case-control status, and the chip batch were used as covariates to adjust the differential methylation analysis. Moreover, surrogate variables analysis (SVA) was applied in order to correct for potential unmeasured or unmodeled confounders. At site level, no statistical differences in methylation between cases and controls with a genome-wide approach (*p* < 10^− 7^) emerged. A list of the top 50 ranked probes as differently methylated is reported in Additional file 2: Table S1. Differential methylation analysis performed at regional level was conducted considering the CpG islands, promoters, genes, and tiling. After multiple testing correction, no statistical differences in region methylation levels emerged between cases and controls. QQ plots showing the level of inflation factor for adjusted and not adjusted analyses are reported in Additional file 3: Figure S2.

### Comparative analysis of differences in methylation status with previous EWAS

The differential methylation analysis is based on the assumption that differences in methylation status are shared among subjects. We have thus evaluated the consensus among previous EWAS reporting differentially methylated genes in the cord blood between in vivo- and in vitro-conceived babies. The results presented with the Venn diagram in Additional file 4: Figure S3 showed a lack of consistency among the studies, with only 6 out of 214 genes found to be differentially methylated in at least two different studies (*NAP1L5*, *L3MBTL*, *GNAS*, *PEG10*, *PRCP*, and *RUNX3*). Importantly, none of these genes were differentially methylated between cases and controls in the present study, despite considering both adjusted and unadjusted *p* values in the regional analysis conducted for genes and promoters. Considering all the 214 genes reported in the literature, only *RNF185* [10] was found in our study to include one of the top 50 ranked probes reported in Additional file 2: Table S1. However, no enrichment of differentially methylated probes was observed for the gene *RNF185* in our population, with only a single probe out of eight associated with the gene resulting to be nominally significant. Additionally, no differences were detected in the regional analysis for genes and promoters (unadjusted *p* values: 0.09 and 0.19 for gene and promoter, respectively).

### Stochastic epigenetic variations analysis

**Fig. 3:**
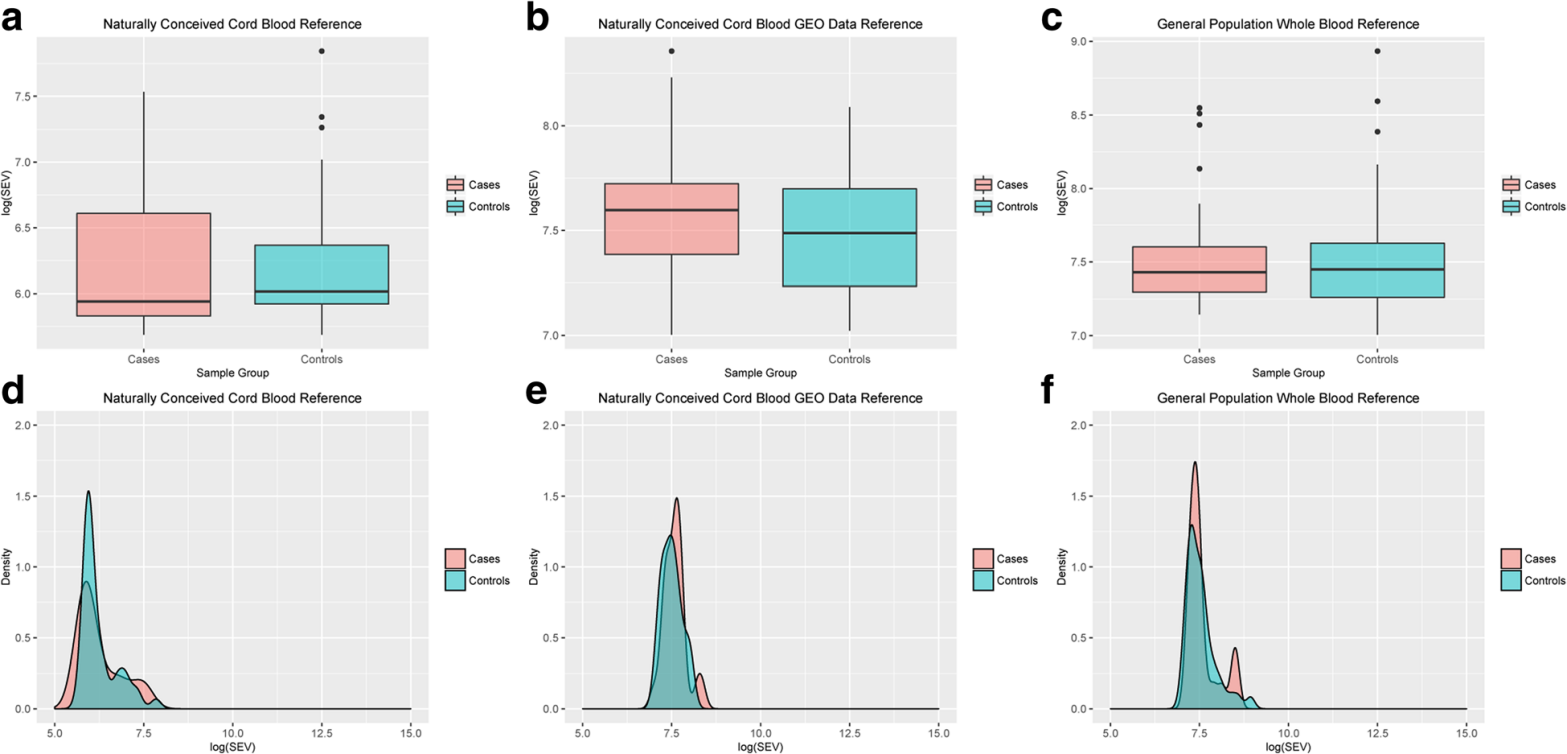
SEVs and ART. For each subject, the total number of SEVs was calculated using three different reference populations. Differences between cases and controls in the number and distribution of SEVs are shown. In panels **a** and **d**, SEVs were computed using naturally conceived cord blood population as reference. In panels **b** and **e**, SEVs were computed using naturally conceived cord blood population obtained from GEO database as reference. In panels **c** and **f**, SEVs were computed using general population whole blood as reference. Number of SEVs is reported in logarithmic scale. Outer limits of the box represent the interquartile range, while the outer limits of the whiskers represent values equal to Q1 – (3 × IQR) and Q3 + (3 × IQR). The central line in each box represents the median number of SEVs

For each subject, the total number of SEVs was calculated using three different reference populations as described in the “Methods” section. The three different estimations of the number of SEVs were then reported using a logarithmic scale and indicated as log(SEVs). The median number of log(SEVs) that was calculated using the naturally conceived cord blood reference was 5.9 (Q1 = 5.8; Q2 = 6.6) in cases (in vitro-conceived babies) and 6.0 (Q1 = 5.9; Q2 = 6.3) in controls (in vivo-conceived babies) (Fig. 3a). The median number of log(SEVs) calculated using the naturally conceived cord blood GEO data reference was 7.5 (Q1 = 7.3; Q2 = 7.7) in cases and 7.4 (Q1 = 7.2; Q2 = 7.6) in controls (Fig. 3b). The median number of log(SEVs) calculated using the general population whole blood reference was 7.4 (Q1 = 7.2; Q2 = 7.6) in cases and 7.4 (Q1 = 7.2; Q2 = 7.6) in controls (Fig. 3c). For all the estimations, no statistically significant differences in log(SEVs) emerged between the two groups analyzed. Moreover, the distribution of log(SEVs) was very similar in cases and controls as shown in the densitograms in Fig. 3d–f. A multivariate logistic regression confirmed that no association was present between log(SEVs) and case-control status after adjustment for the set of covariates used in the differential methylation analysis. Three different regression models were performed according to the three estimations of SEVs, and the results were finally combined using the Fisher’s method. After multiple testing correction, Fisher’s combined *P* value was equal to 0.38. An enrichment analysis was then conducted in order to identify, in each subject, the number of genomic regions that were enriched in SEVs. No differences between ICSI and naturally conceived babies were detected in the number of regions with enriched number of SEVs (Additional file 5: Figure S4A–C). Also the distribution of the number of regions enriched in SEVs number was similar between the two groups. (Additional file 5: Figure S4D–F).

### Stochastic epigenetic variations and imprinting defects

We have also checked for the presence of regions enriched in SEVs in imprinted loci. The list of genes and genomic coordinates under imprinting was selected based on the paper by Court et al. [20]. The analysis identified the presence of a single subject with an enrichment of SEVs located at several differentially methylated regions (DMRs) in the locus *GNAS* (Additional file 6: Figure S5). In this subject from the control group, the high number of reported SEVs suggested a defect in the establishment or maintenance of methylation imprints. The epigenetic defect was confirmed using the methylation-specific multiplex ligation-dependent probe amplification (MS-MLPA) (data not shown).

### Stochastic epigenetic variations association analysis with phenotypic, behavioral, and environmental traits

A multivariate regression analysis was performed in order to identify phenotypic traits potentially associated with SEVs. Three different regression models were performed according to the three estimations of SEVs and the results were finally combined using Fisher’s method. Each multivariate regression model was created considering the logarithmic number of SEVs as dependent variable and the first 10 dimensions obtained from the phenotypic multiple factor analysis as independent variables. Results were adjusted considering the chip batch effect. The multivariate regression showed that the Dim 1, Dim 4, Dim 5, and Dim 9 were strongly associated with the number of SEVs. After multiple testing correction, Fisher’s combined *p* values were *p* = 4.0 × 10^− 4^, *p* = 4.0 × 10^− 4^, *p* = 1.3 × 10^− 3^, and *p* = 5.0 × 10^− 4^, respectively. The phenotypic traits captured by those dimensions are shown in the correlogram in Fig. 1b. Birthweight centiles, gestational age, cesarean section, presence of adverse pregnancy outcomes, pre-gestational mother’s weight, parents’ BMI and obesity, and cord blood cells composition (CD8T, CD4T, and B Cells) were significantly correlated to Dim 1 (*p* < 0.01, ρ > | 0.5 |). Birthweight centiles, mother’s smoking status were significantly correlated to Dim 4 (*p* < 0.01, ρ > | 0.5 |), cord blood cells composition (NK cells) and season of birth were significantly correlated to Dim 5 (*p* < 0.01, ρ > | 0.5 |) while Dim 9 captured mainly cesarean section and pre- or post-conception folates supplementation (*p* < 0.01, ρ > | 0.4 |). The four most significant traits observed to be correlated with SEVs (pre or post conception folates supplementation, cesarean section, mother’s obesity status and season of birth) are shown in Additional file 7: Figure S6. The number of SEVs reported has been obtained using the naturally conceived cord blood reference.

## Discussion

We report the most comprehensive multifactorial analysis of cord blood DNA methylation of in vitro conceived babies and the first analysis of the stochastic variations potentially induced by ART techniques. Although the use of ART has allowed millions of otherwise infertile couples to conceive children, some concern still remains about the safety of these procedures [2, 21]. An increase in imprinting disorders has been found in children conceived through IVF and ICSI, but no evidence of generalized changes in DNA methylation could be appreciated [2]. Our results are in line with this observation based on two different approaches: (i) a conventional epigenome-wide association analysis. At both site and region levels, no differences in methylation status between naturally and in vitro conceived emerged as statistically significant; (ii) a previously published method aimed at investigating the number and the localization of stochastic epigenetic variations [12, 22].

Although several studies have tried to investigate the methylation status of ART-conceived babies, only four EWAS were previously conducted on this topic. Melamed et al. have evaluated 27,578 CpG sites in cord blood from 10 children conceived in vitro and 8 conceived in vivo [10]. Castillo-Fernandez et al. investigated the links between IVF and DNA methylation patterns in 47 IVF and 60 non-IVF newborn twins (from 54 twin pairs) in whole cord blood cells and cord blood mononuclear cells using genome-wide methylated DNA immunoprecipitation coupled with deep sequencing [13]. Estill et al. reported the methylation profiles of neonatal blood spots of 137 babies conceived naturally or with four different ART techniques [14]. Finally, El Hajj N et al. evaluated the methylome of 48 babies conceived with ICSI [15]. Both Estill et al. and El Hajj N et al. used the Infinium HumanMethylation450 BeadChips to analyze the methylation levels [14, 15]. Notwithstanding the lack of an adequate correction for crucial confounding factors, only a small number of CpG sites was differentially methylated at a genome-wide level and the magnitude of differences identified was also extremely small [10, 13–15]. Furthermore, when considering all the studies together, we observed a substantial inconsistence among their results. Only six genes have been confirmed in at least two studies (*NAP1L5*, *L3MBTL*, *GNAS*, *PEG10*, *PRCP*, *and RUNX3*).

Interestingly, a review of literature concerning those genes revealed that they are already known to be epigenetically associated with other pregnancy conditions. *NAP1L5*, for example, has been previously identified to be differently methylated in cord blood samples characterized by intrauterine exposure to gestational diabetes mellitus [23]. *L3MBTL* has been reported to be differently methylated in cord blood of babies exposed to smoking during pregnancy [24] and is epigenetically associated with gestational age [25]. Increased methylation at differentially methylated regions of *GNAS* has been described in infants born in conditions of gestational diabetes [26]. An increase in DNA methylation at the *SGCE/PEG10* DMR has been previously positively associated with paternal BMI [27], maternal stress [28], and also with maternal prenatal physical activity [29]. Methylation of *PRCP* gene in newborns [30] as well as *RUNX3* [24] was found to be significantly associated with maternal smoking status. Hypermethylation of *RUNX3* CpG sites has been also associated with decreased gestational age [24]. Taken together, this evidence hints at the presence of confounding factors in previously reported results on epigenetics of ART. This observation seems to be supported by analysis of the QQ-plot obtained from unadjusted analysis presented herein. The elevated genomic inflation factor (lambda = 1.7; Additional file 3: Figure S2) observed in unadjusted analysis sheds light on the excess of false positive rate potentially produced under inadequate correction. The influence of confounding factors on epigenome of cord blood has been extensively described. Soubry et al., for example, demonstrated a significant association between parental BMI and DNA methylation of imprinted genes [29]. This finding should be considered as relevant since a significant effect of ART has been reported to affect imprinted loci [15].

An increased variability of DNA methylation in IVF conceived babies was previously underlined, hinting at the presence of epigenetic changes not shared among subjects [10] and thus supporting the idea that the effect of ART might be more likely to be stochastic rather than confined to some genetic regions. Our novel analytical approach addressing the number of stochastic epigenetic variations [12, 22] showed no differences in cord blood from in vitro and in vivo conceived babies.

The analysis of SEVs proposed in the present study proved to be a powerful approach to study the epigenetic variability and allowed to study the impact of several conditions on the newborn epigenome. Interestingly, four dimensions obtained from the dimensional reduction of phenotypic traits were significantly associated with the number of SEVs. Cord blood cell composition, pre or post conception supplementation of folates, birth percentiles, gestational age, cesarean section, pre-gestational mother’s weight, parental BMI and obesity, presence of adverse pregnancy outcomes, mother’s smoking status, and season of birth were the traits mainly captured by these dimensions. Some of these factors such as parental BMI, gestational age, birth weight, and smoking are already known for playing a role on the newborn epigenome [20, 25, 27–29, 31]. Of note, we observed and reported for the first time the existence of an epigenetic signature associated with season of birth. The number of stochastic epigenetic variations was lower in subjects born in autumn. This observation should be considered important for at least two reasons: (i) seasonality of birth has already been reported to be associated with an increased incidence of several pathological conditions such as type I diabetes [32, 33], cardiovascular disease [34], skin cancer [35], and autoimmune diseases [36]. Moreover, seasonality of birth has been also studied in the field of aging and is associated with life expectancy [37–40]; (ii) the magnitude of the effect of seasonality, a natural event, on epigenome of newborns appeared to be greater compared to the one induced by ART.

We have also evaluated the presence of SEVs in imprinted loci failing to find significant differences between cases and controls. A single epigenetic alteration in the locus *GNAS* was found in a control subject. It is important to note that the new method applied in this study allowed not only to evaluate the number of SEVs located across the whole genome, but also succeeded in the identification of a single epigenetic alteration subsequently confirmed using MS-MLPA and not shared among subjects. A standard case-control analysis would have failed to identify this particular defect. To our knowledge, this is the first study addressing a genome-wide analysis of imprinted loci taking into account the probable random effect of ART and applying a test that does not require the possible epigenetic variation to be present in more than one subject. In any case, we realize that these phenomena do represent rare events, and we probably need a greater sample size to completely exclude a direct effect at the level of specific imprinting genes.

The present work has clarified that the number of stochastic epigenetic variations potentially induced by ART technology was, at worst, comparable to that naturally produced in response to maternal behavior or other common environmental factors, thus debunking the idea of a severe impact of ART in the epigenome of the newborns and implying a reconsideration of the epigenetic safety of these techniques.

Another crucial aspect of the present work is the analysis of all the potential confounders that could affect the methylation status in the newborn cord blood. It is totally unexpected that previous studies on the same topic evaluating genome wide DNA methylation [10, 13–15] failed to correct for factors potentially affecting methylation status or considered only a small number of them. Therefore, given the complexity of the networks and of the phenotypic traits involved in pregnancy establishment, the causal relationship between the epigenetic status and ART needs to be evaluated with caution and controlling for potential confounders. The complexity of the phenotypic traits represents also another important issue; the correlation analysis performed among phenotypic traits revealed the existence of several hidden associations among variables, and this needs to be considered when adjusting for confounders in order to avoid multicollinearity.

Another aspect that is often disregarded is the potential role of ART techniques on placenta methylation status and the consequent effect on birth weight and on the epigenome of newborns. Recent studies conducted in animal models reported that ART has a predominant or exclusive effect on the placenta methylation status compared to that on the fetus [9, 41, 42]. An attractive hypothesis that comes from these data is that the higher incidence of premature birth and low birth weight observed in ART-conceived children may be related to abnormal placental function resulting from genomic imprinting errors at multiple genes. Recently, Ghosh et al. reported evidence of placental methylation altered by ART procedures at repeated sequences (LINE1 elements) and CCGG sites [43]. Similarly, Choux et al. reported lower DNA methylation for two imprinted loci (*H19/IGF2* and *KCNQ1OT1* DMRs) and two transposon families (LINE-1Hs and ERVFRD-1) in the placenta of babies conceived by ART [44]. Interestingly, DNA methylation of the same imprinted genes DMRs and transposable elements in cord blood was not altered by ART procedures, thus confirming the main effect at the placental level as seen in animal experiments. A whole genome analysis of the methylation status of placenta samples from babies conceived by ART is needed in order to elucidate this crucial aspect.

A number of limitations need to be considered when interpreting our results. First, only DNA from cord blood was analyzed. Since cord blood is not necessarily representative of the epigenetic status of all tissues and cells in the newborn, additional studies on other tissues are mandatory to confirm these data. Second, the phenotypic data of the studied subjects did not include all the possible variables related to pregnancy and labor although a surrogate variables analysis was conducted to compensate for this deficiency. Third, although the number of subjects used for comparisons is of the same order of magnitude of previously conducted EWAS, we must underline that a larger sample size could better estimate normal ranges of DNA methylation in each locus. Confirming our results on a larger sample size would allow to exclude the possibility that small methylation differences have gone undetected. Fourth, these findings represent the experience relative to the ICSI procedure of a single IVF center and a single lab. Although we selected only ICSI procedures in order to analyze a homogeneous group of babies obtained using the more invasive technique, we cannot exclude that procedures done in different ways and with different technologies could result in different data. Moreover, it should be considered that when comparing naturally conceived and in vitro-conceived babies, there is a consistent number of inescapable differences between the two groups such as parents’ infertility status that may represent an important selection bias. Finally, although the Infinium HumanMethylation450 BeadChips covers 99% of RefSeq genes, permitting a whole-genome analysis, the coverage of total CpG sites is low (around 2%). This means that sequencing-based methods should be adopted in the future in order to study other genomic features (e.g., enhancers) that have only small coverage in the present analysis.

## Conclusions

The presented prospective study does not support that children born after ART have an increased risk of genome-wide changes in DNA methylation neither at specific loci neither randomly scattered throughout the genome. On the other hand, the study confirms that there are several environmental and behavioral conditions able to affect epigenetic variability in cord blood and leads to the conclusion that they need to be considered as potential confounders in investigations of this nature.

A reanalysis of previous data based on phenotypic traits of the parents and the babies potentially associated with epigenetic changes is warranted as well as a meta-analysis including all the data from genome-wide studies.

## Methods

### Study design and study population

This is a prospective study designed to avoid biases related to the improper selection of complicated pregnancies in one of the two groups. Women that underwent ICSI treatment were enrolled in the study at 20 weeks’ gestation. These women were stimulated with standard ovulation induction drugs. Pregnant women who naturally conceived were also enrolled at 20 weeks’ of gestation. The in vivo group had no history of infertility, and the index pregnancy was achieved without medications or treatments. None of the pregnancy ended in abortion, and all the patients were included in the study. Samples of cord blood from both ART-conceived pregnancies (*n* = 23) and naturally conceived pregnancies (*n* = 41) were obtained at the time of delivery by the midwives of the San Raffaele Hospital, Milano, Italy, by puncturing the umbilical vein while the placenta was in utero [45]. Patients were informed that cord blood would be used for research purposes and gave written consent. Approval for this study was granted by the local Human Institutional Investigation Committee (#PMAMET). Clinical information obtained for each pregnancy included demographic and obstetric factors, cause of infertility, details of the ART procedure as well as pregnancy, delivery, and neonatal outcomes. Multifetal gestations were excluded from both groups.

Sample size was calculated based on the analysis of parameters observed in a previous study in which more than 400 subjects were evaluated [12]. Mean and variance were estimated for all the CpG sites of the Infinium HumanMethylation 450 BeadChip array. The number of subjects to be enrolled has been calculated assuming an effect size taking into account a difference in the percentage of methylation between groups of at least 10%, by imposing a probability of type I error in the order of 10^− 7^ (level of significance that takes into account the need to correct for multiple testing) and a power of 95%. A description of the study design is shown in Additional file 8: Figure S7.

### In vitro fertilization procedures

Controlled ovarian stimulation was performed according to the clinical practice and as previously described [46–48]. Oocyte collection was performed 36 h after triggering of ovulation. After 2–3 h incubation in Human Serum Albumin (HSA)-supplemented Fertilization medium (Sage In-Vitro Fertilization, Inc. Trumbull, CT, USA) under oil, denudation of the cumulus oophorus was performed as previously described [46, 49, 50]. Injected oocytes were grouped-cultured in microdrops of equilibrated Serum Substitute Supplement (SSS, Irvine, CA, USA)-supplemented Cleavage medium (Sage In-Vitro Fertilization, Inc. Trumbull, CT, USA) under oil. Sixteen to 18 h after ICSI, all oocytes were checked for fertilization as previously described [46, 49, 50]. For a subgroup of patients (*n* = 7, 30.4%), embryos were cultured to blastocyst stage in SSS (Irvine, CA, USA)-supplemented Blastocyst medium (Sage In-Vitro Fertilization, Inc. Trumbull, CT, USA). All the incubation steps were conducted using low (5%) oxygen concentration incubators [49]. All the transfers were performed in fresh cycles [48]. All the patients underwent luteal phase support with progesterone 600 mg/d (Prometrium) administered vaginally and continued through week 12 of pregnancy.

### DNA extraction and bisulphite treatment of the DNA

Genomic DNA was extracted from cord blood using the Wizard genomic DNA purification kit (PROMEGA, Madison WI, USA) as previously described [12]. Quality control and quantification of DNA were performed before and after bisulphite conversion. DNA was quantified with NanoDrop (NanoDrop Products Thermo Scientific, Wilmington, DE, USA) and quality was assessed by visualization of genomic DNA on 1% agarose gel electrophoresis. Only DNA samples not fragmented and with a concentration higher than 50 ng/μl were subsequently processed.

### DNA methylation assay

4 μl of bisulfite-converted DNA was used for hybridization on Infinium HumanMethylation 450 BeadChip, following the Illumina Infinium HD Methylation protocol. This consists of a whole genome amplification step followed by enzymatic end-point fragmentation, precipitation, and resuspension. The resuspended samples were hybridized on HumanMethylation 450 BeadChips at 48 °C for 16 h. Then, unhybridized and non-specifically hybridized DNA were washed away followed by a single nucleotide extension using the hybridized bisulfite-treated DNA as a template. The nucleotides incorporated were labeled with biotin (ddCTP and ddGTP) and 2,4-dinitrophenol (DNP) (ddATP and ddTTP). After the single base extension, repeated rounds of staining were performed with a combination of antibodies that differentiate DNP and biotin by fixing them different fluorophores. Finally, the BeadChip was washed and protected in order to scan it. The Illumina HiScan SQ scanner is a two-color laser (532 nm/660 nm) fluorescent scanner with a 0.375 μm spatial resolution capable of exciting the fluorophores generated during the staining step of the protocol. Image intensities were extracted using GenomeStudio (2010.3). The methylation score for each CpG site was represented as β values according to the fluorescent intensity ratio between methylated and unmethylated probes. β values may range between 0 (unmethylated) and 1 (completely methylated).

### Data management, pre-processing, normalization, and quality control

Illumina Methylation 450K raw data were analyzed using the RnBeads analysis software package [51]. Sites with overlapping SNPs were firstly removed from the analysis (*n* = 4713) as well as probes on sex chromosomes (*n* = 11119). Possible removal of probes and samples of highest impurity from the dataset was evaluated using the Greedycut algorithm. We considered every β value to be unreliable when its corresponding detection *p* value was not below the threshold (*T* = 0.05). In order to avoid an erroneous interpretation of stochastic epigenetic variations, probes with coordinates overlapping rare genetic variants annotated in 1000 genomes and EXAC databases were removed [52, 53]. After the quality control step, none of the samples was excluded for quality reasons while a total of 14208 probes were removed. The background was subtracted using the methylumi package (method “noob”) [51]. The signal intensity values were normalized using the SWAN normalization method, as implemented in the minfi package. In addition to CpG sites, four sets of genomic regions were covered in the analysis (tiling, genes, promoters and CpG Islands).

### Blood cell type counts

Proportions of CD8 T cells, CD4 T cells, NK cells, B cells, monocytes, and granulocytes were estimated using the “estimateCellCounts” function in the Bioconductor minfi package [54] with the reference data for cord blood provided by Bakulski et al. [55].

### Differential methylation analysis

Differential methylation analysis was conducted both at site and region level according to the sample groups. *p* values were computed using the limma method for the site level analysis while a combined *p* value was calculated from all site *p* values for the region-based [51]. Regions were defined according to RnBeads definitions [51]:

- Genes and promoters: Ensembl [56] gene definitions were downloaded using the biomaRt package. A promoter was defined as the region spanning 1500 bases upstream and 500 bases downstream of the transcription start site of the corresponding gene.
- GpG islands: the CpG island track was downloaded from the dedicated FTP directory of the UCSC Genome Browser [57].
- Tiling regions: not overlapping tiling regions with a window size of 5 kb were defined over the whole genome.

In order to avoid potentially confounding factors, a multiple factor analysis (MFA) was performed based on phenotypic data (birth weight, birthweight centiles, mother’ s age, parity status, gestational age, cesarean section, sex of the baby, presence of adverse pregnancy outcomes, pre-gestational mother weight, parents’ BMI and obesity status, gestational weight increase, smoking status, pre-post conception folate supplementation, CD8T, CD4T, NK cells, B cells, monocytes, granulocytes, season of birth). Variables were entered in the analysis as factorial or linear values. Chip batch and dimensions that were significantly associated with the case-control status were used as covariates in the differential methylation analysis. Adjustment for surrogate variables was conducted using the function directly provided in the package RnBeads, which can detect batch effects and other unwanted variation of unknown origin and annotate them in such a way that they can be controlled for as covariates [51].

### Epigenetic variations detection

In order to identify stochastic epigenetic variations (SEVs), we used a method previously described by our group [12, 22]. Briefly, after the pre-processing step, the distribution and variability of methylation levels were studied in the populations for all the probes of the array using box and whiskers plots. At each CpG site, the methylation level of a subject that was extremely different from the rest of the population was counted as an epigenetic variation. Thus, for each locus, epigenetic variations were identified as the extreme outliers, with their methylation level that lied outside of Q1 − (3 × IQR) and Q3 + (3 × IQR). Finally, all the observed epigenetic variations were annotated in a new data matrix that allowed to calculate, for each subject, the total amount of epigenetic variations and their genomic position. The box and whiskers plot analysis was conducted using boxplot function in the R car package and confirmed using the outlier function in the R outliers package.

In addition to the previously described control population composed by 41 natural conceived babies, two other different reference populations have been used for this analysis:

1. Methylation row data from 60 subjects were obtained from the public functional genomics data repository GEO. We have selected two different datasets containing methylation data from cord blood samples (GSE54399, GSE30870), and we have extracted only methylation data from control subjects. This population (naturally conceived cord blood GEO data reference) represented our “external” control population. GEO and in house raw data were pre-processed following the same procedure.
2. Methylation row data from 350 healthy subjects with an age spanning from 1 to 107 years were also obtained from the Istituto Auxologico Italiano Epigenetic Database. This population represented a second reference population used for the estimation of epigenetic variations (general population whole blood reference).

A schematic description of the strategy used to obtain different estimations of the number of SEVs by using the various reference populations is shown is Additional file 9: Figure S8.

Briefly:

- In the first step, the analysis described above was applied on naturally conceived cord blood population. Samples were analyzed together, and the number of SEVs was calculated in each control subject.
- In a second step, all the samples in the case group were tested individually using the naturally conceived cord blood reference and the number of SEVs was calculated for each case subject.
- In the third step, the naturally conceived cord blood GEO data reference was used to calculate the number of SEVs both in cases and control subjects. Also in this step, each subject was tested individually.
- Finally, the general population whole blood reference was used to calculate the number of SEVs both in control and case subjects. Also in this step, each subject was tested individually.

Using three different reference populations, three different estimations of the number of SEVs were calculated for each subject. A test for over-representation of these probes was conducted, for each subject, using sliding windows and the hypergeometric cumulative function, obtaining the number of genomic regions that were enriched in SEVs [12].

### Validation of the SEVs analysis

In order to confirm the power of this analytical approach to detect epigenetic variations, two separate tests were performed on positive controls. Three samples were analyzed in duplicates, and epigenetic variations found in each of them were compared. Results showed a mean correlation of 0.99 (*p* < 0.01) among the experiments. The duplicate samples underwent independent bisulfite conversion reactions, and this suggests that epigenetic variations are not significantly influenced by bisulfite conversion errors. In the second validation step, 48 whole blood DNA samples obtained from subjects affected by imprinting diseases (Beckwith-Wiedemann syndrome, Angelman syndrome and Silver Russel syndrome) who underwent diagnostic assays at Istituto Auxologico Italiano were analyzed. For these subjects, a medical report indicating the genomic position of their epigenetic alteration was already available. Briefly, after the identification of the outlier probes, a test for over-representation of these probes inside each gene was performed using the hypergeometric cumulative function. The analysis identified genes with enriched number of outliers probes (Bonferroni’s corrected *p* value < 0.05) confirming the presence of the epigenetic alterations previously reported in the medical report.

### Statistical tests

The “Shapiro.test” function provided in the R package “stats” was applied to test normality among variables. The “Wilcox.test” function provided in the R package “class” was used to test differences between cases and controls groups for all non-parametric data. Considering the presence of categorical variables, dimensional reduction was performed using the multiple factor analysis of mixed data approach and the “FAMD” function provided in the R package “FactoMineR.” The univariate and multivariate linear regressions were conducted using the “Generalised Linear Model” function provided in the R “base” package. Bonferroni’s correction was performed to correct for multiple testing. Correlation analysis between Dims and environmental and behavioral conditions has been performed using the hector function provided in the package “polycor.”

### Comparative analysis with previous EWAS

We conducted a systematic search of the literature focusing on studies that used a genome-wide approach (Illumina Infinium HumanMethylation27 BeadChip and Illumina Infinium Human Methylation 450K BeadChip) with the aim to compare cord blood methylation levels between in vitro and in vivo conceived babies. The four selected studies [10, 13–15] reported differentially methylated genes using different parameters for the selection. Melamed et al. identifies genes that had at least two significantly differentially methylated CpG sites and genes with at least a CpG site showing a significant methylation difference ≥ 10% between ART and control groups [10]. A total of 33 genes were found. Estill et al. considered as significant all those genes with a minimum absolute average methylation change of 2.5% for the clusters of CpGs that were associated with a given gene (at least 75% of the cluster intersecting a gene body or promoter), with only the significant clusters that were considered in the average count [14]. The study reported a list of differentially methylated metastable epialleles, imprinted genes, and genes related to differentially methylated enhancers. A total of 87 genes as having significantly different methylation status emerged when comparing babies born with various ART techniques and naturally conceived. Castillo-Fernandez et al. reported a list of all the genes located near each differentially methylated CpGs between the two groups selected using a false discovery rate equal to 25% [13]. A total of 66 genes were found to be associated with the 46 reported differentially methylated regions. Finally, El Hajj et al. reported a list of 34 genes with a methylation difference of β > ±0.03 and an adjusted *p* < 0.05 observed in promoters, imprinting control regions and CpG islands [15].

## Additional files

Additional file 1: **Figure S1.** Correlation analysis among phenotypic, behavioral and environmental features considered in the study. Degree and direction of correlations are highlighted by the color and dimension of squares. (TIF 307 kb)

Additional file 2: **Table S1.** Top 50 ranked hypomethylated or hypermethylated probes in babies born after ICSI. (XLS 41 kb)

Additional file 3: **Figure S2.** QQ plots obtained using (A) Dim1 and Dim3 resulted from the multiple factor analysis of mixed data. (B) Surrogate variable analysis (SVA), and Dim1 and Dim3 obtained from the multiple factor analysis of mixed data as covariates in the differential methylation analysis. The QQ plot obtained without data correction is illustrated in C. The elevated genomic inflation factor of unadjusted data suggests presence of potential confounders. (TIF 141 kb)

Additional file 4: **Figure S3.** Venn diagram illustrating the number of genes found in literature to be differentially methylated in cord blood of ART babies when compared to natural conceived babies and the overlapping of results among previous EWAS [10, 13–15]. (TIF 234 kb)

Additional file 5: **Figure S4.** For each subject, the total number of region enriched in SEVs was calculated using three different reference populations. Differences between cases and controls in the number and distribution of region enriched in SEVs are shown. In panels A and D, SEVs were computed using naturally conceived cord blood population as reference. In panels B and E, SEVs were computed using naturally conceived cord blood population obtained from GEO database as reference. In panels C and F, SEVs were computed using general population whole blood as reference. Number of region enriched in SEVs is reported in logarithmic scale. Outer limits of the box represent the interquartile range, while the outer limits of the whiskers represent values equal to Q1 − (3 × IQR) and Q3 + (3 × IQR). The central line in each box represents the median number of SEVs. (TIF 181 kb)

Additional file 6: **Figure S5.** Genomic regions under imprinting control carrying an epigenetic alteration in a control subject. In panel A, stochastic epigenetic variations (SEVs) detected in the cord blood of a single subject from the control population are represented in red while the differentially methylated regions (DMRs) reported by Court et al. [18] are represented in blue. The high number of reported SEVs suggested a defect in the establishment or maintenance of methylation imprints confirmed using MS-MLPA. Panel B illustrates the magnification of one of the DMRs. (TIF 1062 kb)

Additional file 7: **Figure S6.** Effect of folates supplementation, mother’s obesity status, cesarean section, and season of birth on number of SEVs. Number of SEVs is reported in logarithmic scale. Outer limits of the box represent the interquartile range, while the outer limits of the whiskers represent values equal to Q1 – (3 × IQR) and Q3 + (3 × IQR). The central line in each box represents the median number of SEVs. (TIF 107 kb)

Additional file 8: **Figure S7.** Schematic representation of the study design. (TIF 1116 kb)

Additional file 9: **Figure S8.** Schematic description of the strategy used to estimate the number of SEVs. (TIF 982 kb)

## Abbreviation

ART: Assisted reproduction technology
BMI: Body mass index
Dim: Dimension
DMR: Differentially methylated region
EWAS: Epigenome wide association study
ICSI: Intra citoplasmatic sperm injection
IVF: in vitro fertilization
NGS: Next generation sequencing
SEV: Stochastic epigenetic variation
SVA: Surrogate variable analysis
