# Supplementary figures and images for: Multifactorial analysis of the stochastic epigenetic variability in cord blood confirmed an impact of common behavioral and environmental factors but not of in vitro conception
Source: Clin Epigenetics. 2018 Jun 8;10:77. doi: 10.1186/s13148-018-0510-3 (PMC5994106; doi:10.1186/s13148-018-0510-3)

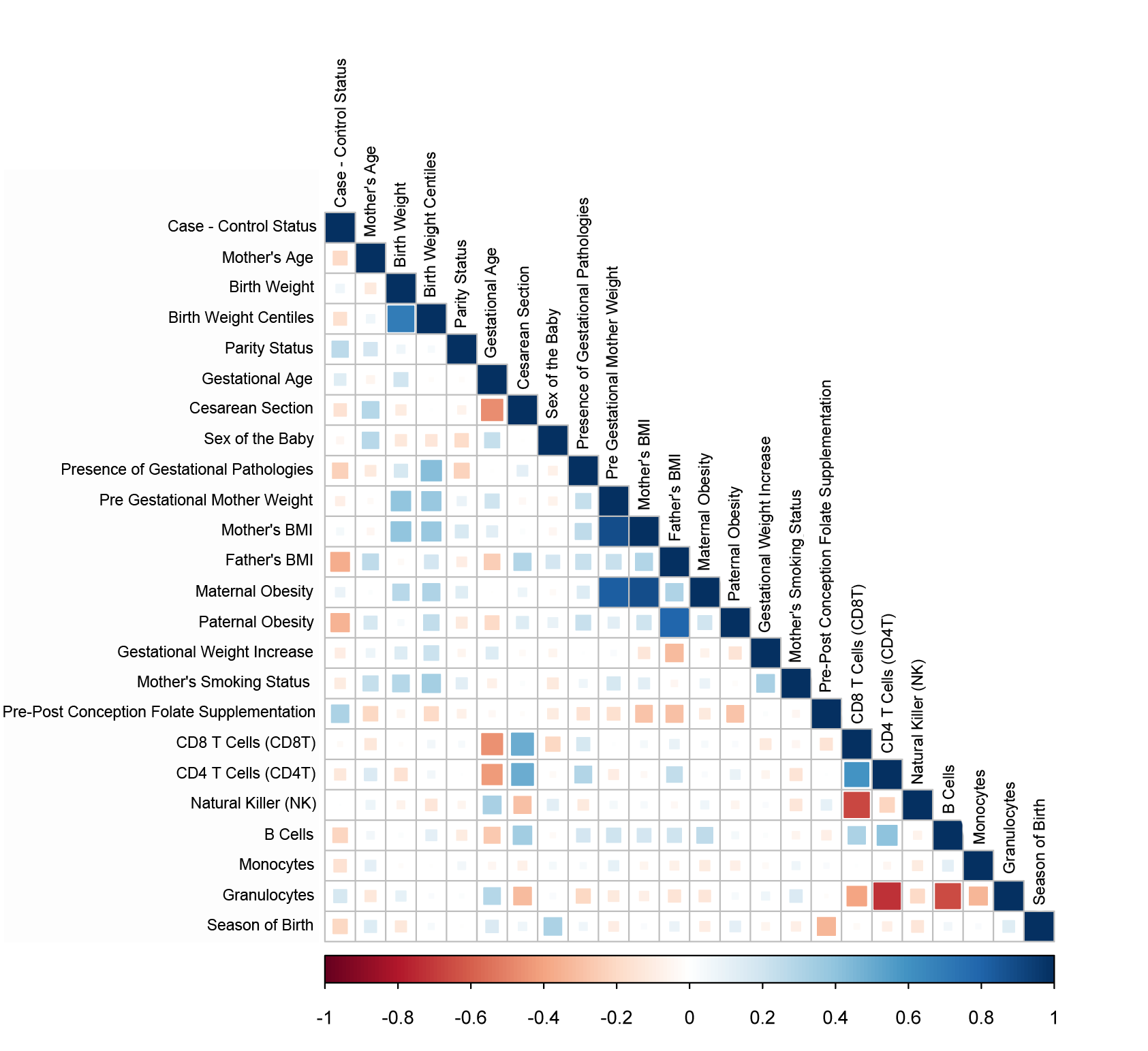

Supplement: Supplementary file 1 — Figure S1. Correlation analysis among phenotypic, behavioral and environmental features considered in the study. Degree and direction of correlations are highlighted by the color and dimension of squares. (TIF 307 kb) [file 13148_2018_510_MOESM1_ESM.tif]

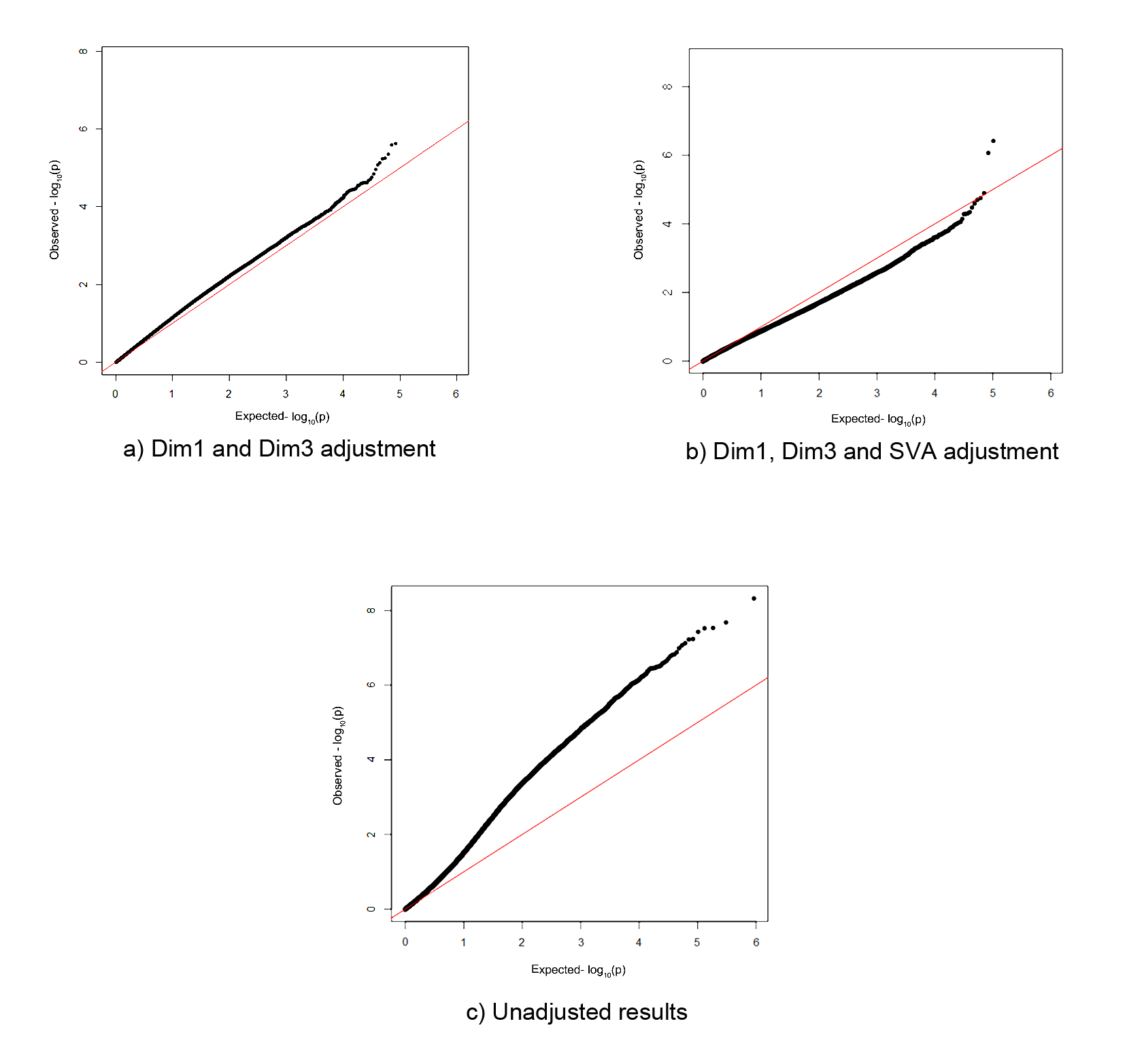

Supplement: Supplementary file 3 — Figure S2. QQ plots obtained using (A) Dim1 and Dim3 resulted from the multiple factor analysis of mixed data. (B) Surrogate variable analysis (SVA), and Dim1 and Dim3 obtained from the multiple factor analysis of mixed data as covariates in the differential methylation analysis. The QQ plot obtained without data correction is illustrated in C. The elevated genomic inflation factor of unadjusted data suggests presence of potential confounders. (TIF 141 kb) [file 13148_2018_510_MOESM3_ESM.tif]

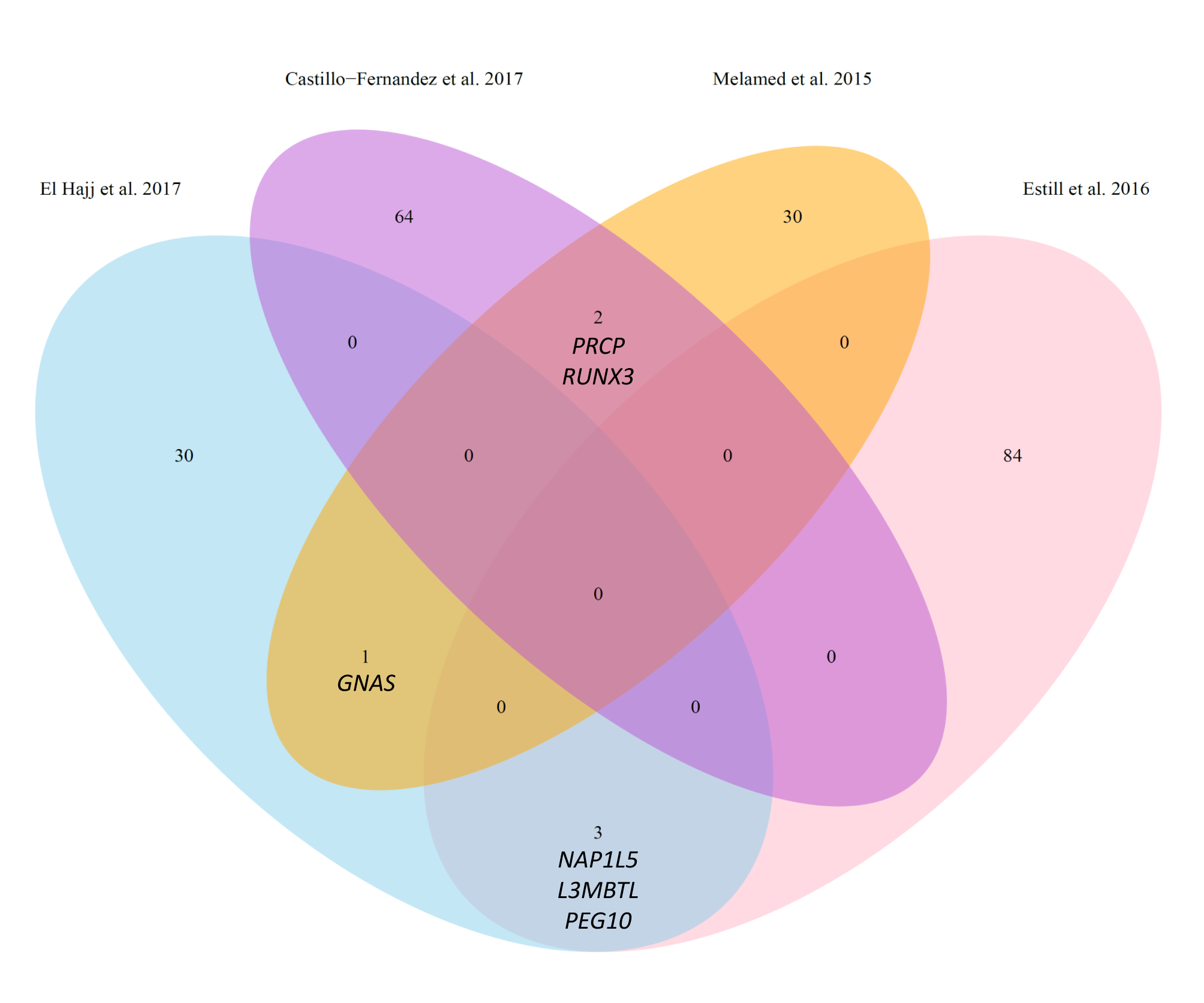

Supplement: Supplementary file 4 — Figure S3. Venn diagram illustrating the number of genes found in literature to be differentially methylated in cord blood of ART babies when compared to natural conceived babies and the overlapping of results among previous EWAS [10, 13–15]. (TIF 234 kb) [file 13148_2018_510_MOESM4_ESM.tif]

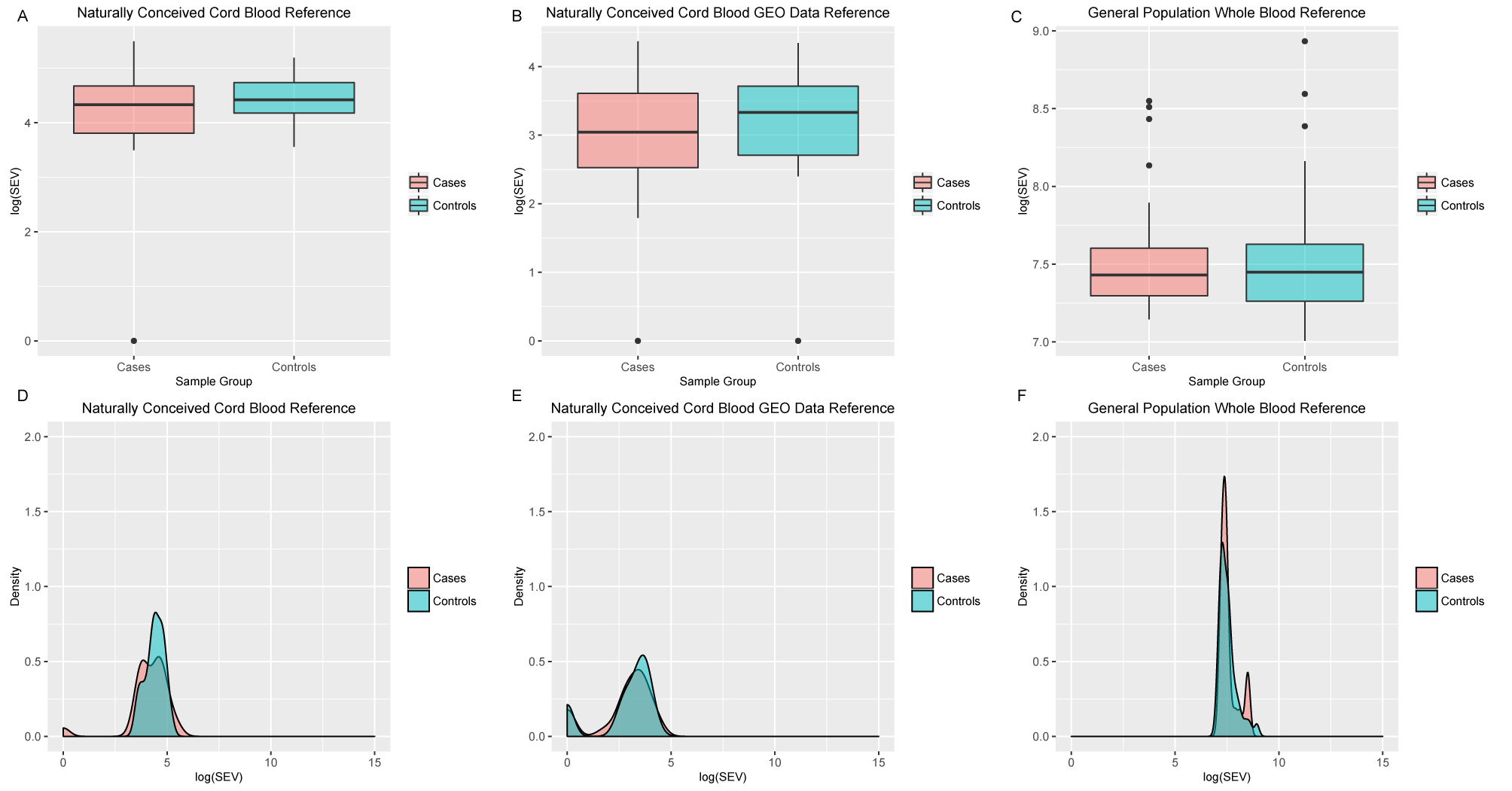

Supplement: Supplementary file 5 — Figure S4. For each subject, the total number of region enriched in SEVs was calculated using three different reference populations. Differences between cases and controls in the number and distribution of region enriched in SEVs are shown. In panels A and D, SEVs were computed using naturally conceived cord blood population as reference. In panels B and E, SEVs were computed using naturally conceived cord blood population obtained from GEO database as reference. In panels C and F, SEVs were computed using general population whole blood as reference. Number of region enriched in SEVs is reported in logarithmic scale. Outer limits of the box represent the interquartile range, while the outer limits of the whiskers represent values equal to Q1 − (3 × IQR) and Q3 + (3 × IQR). The central line in each box represents the median number of SEVs. (TIF 181 kb) [file 13148_2018_510_MOESM5_ESM.tif]

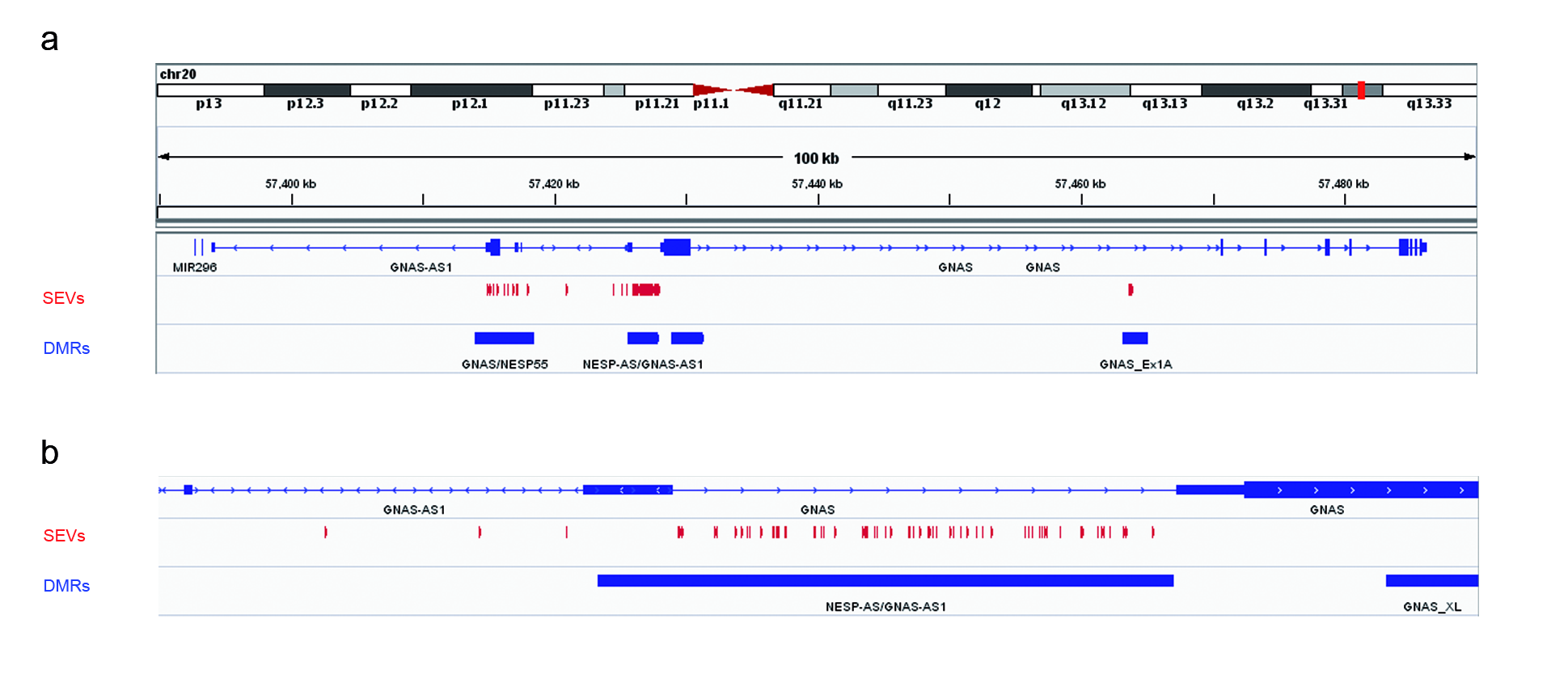

Supplement: Supplementary file 6 — Figure S5. Genomic regions under imprinting control carrying an epigenetic alteration in a control subject. In panel A, stochastic epigenetic variations (SEVs) detected in the cord blood of a single subject from the control population are represented in red while the differentially methylated regions (DMRs) reported by Court et al. [18] are represented in blue. The high number of reported SEVs suggested a defect in the establishment or maintenance of methylation imprints confirmed using MS-MLPA. Panel B illustrates the magnification of one of the DMRs. (TIF 1062 kb) [file 13148_2018_510_MOESM6_ESM.tif]

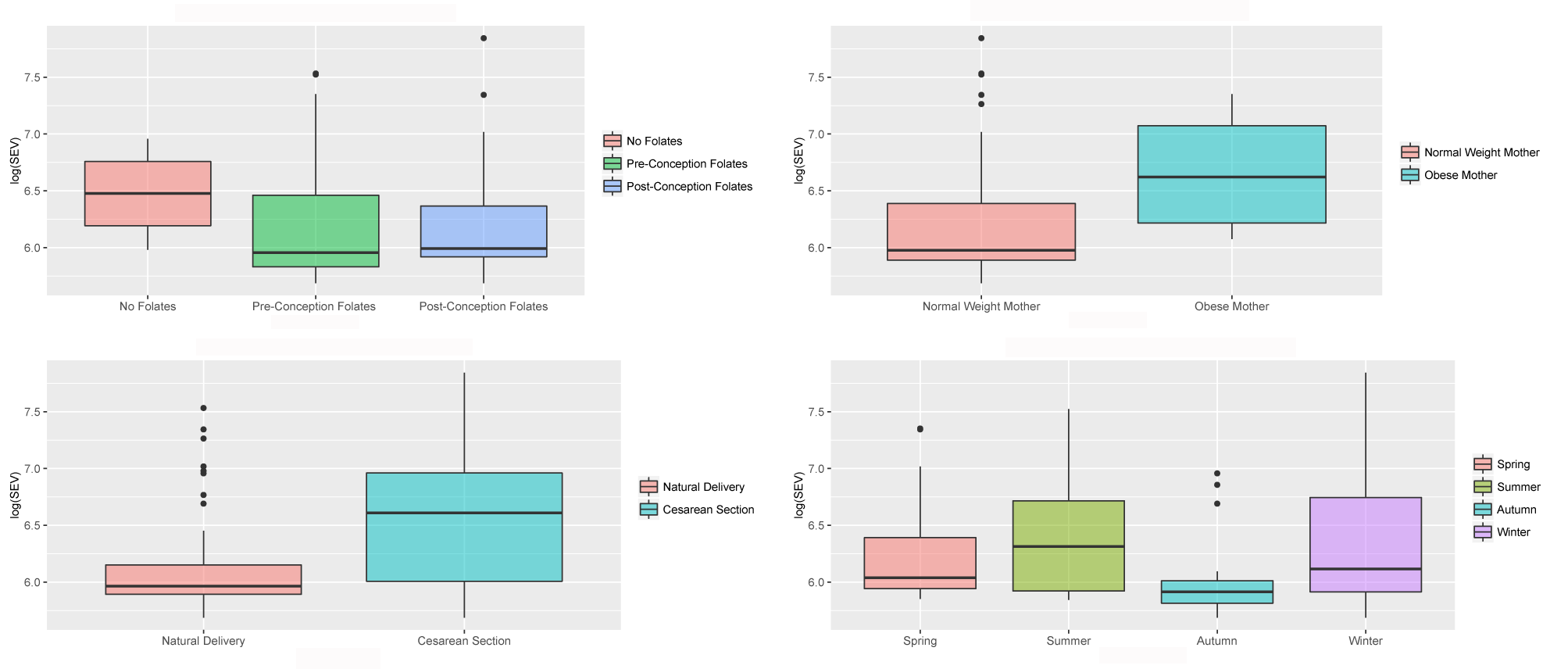

Supplement: Supplementary file 7 — Figure S6. Effect of folates supplementation, mother’s obesity status, cesarean section, and season of birth on number of SEVs. Number of SEVs is reported in logarithmic scale. Outer limits of the box represent the interquartile range, while the outer limits of the whiskers represent values equal to Q1 – (3 × IQR) and Q3 + (3 × IQR). The central line in each box represents the median number of SEVs. (TIF 107 kb) [file 13148_2018_510_MOESM7_ESM.tif]

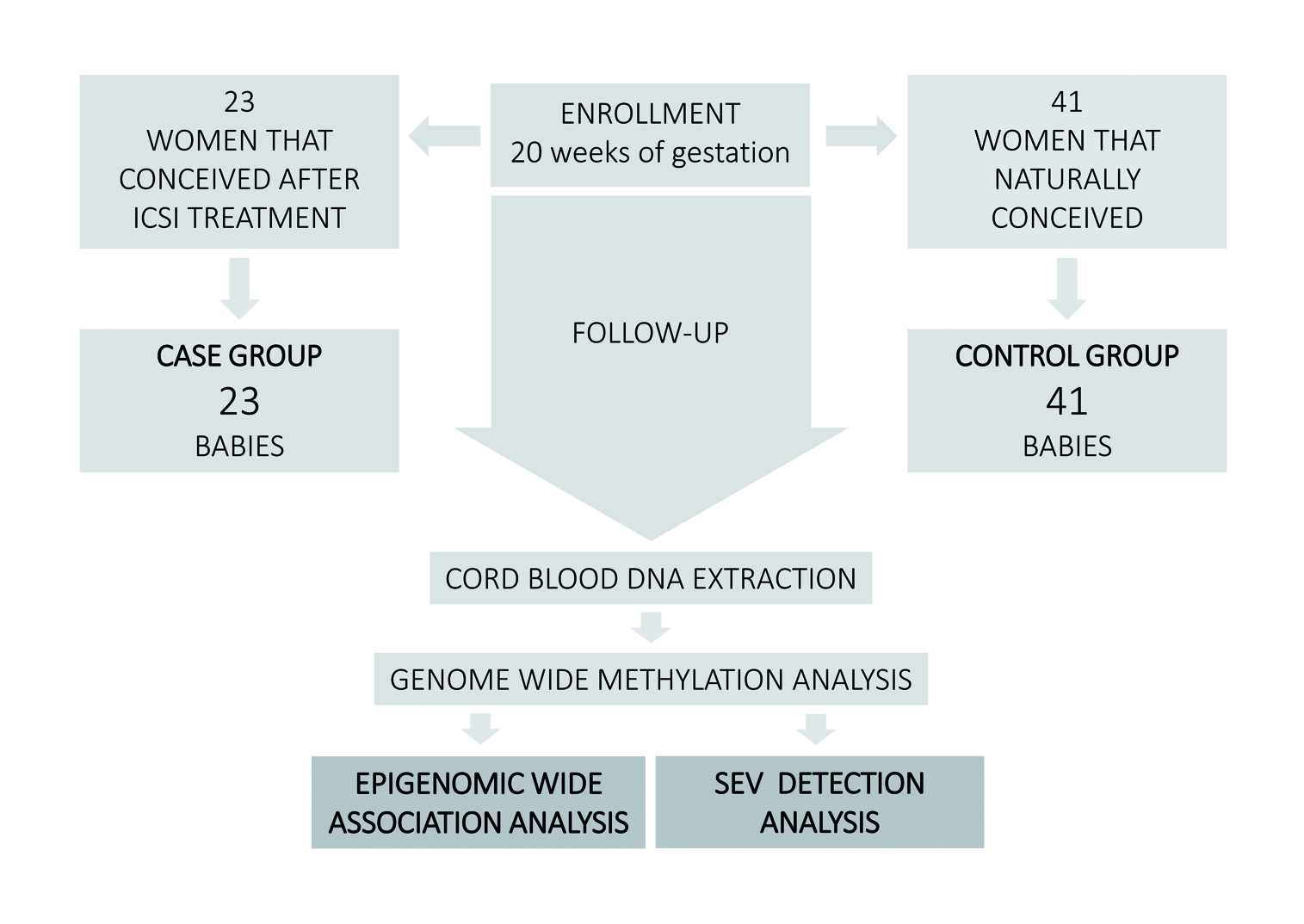

Supplement: Supplementary file 8 — Figure S7. Schematic representation of the study design. (TIF 1116 kb) [file 13148_2018_510_MOESM8_ESM.tif]

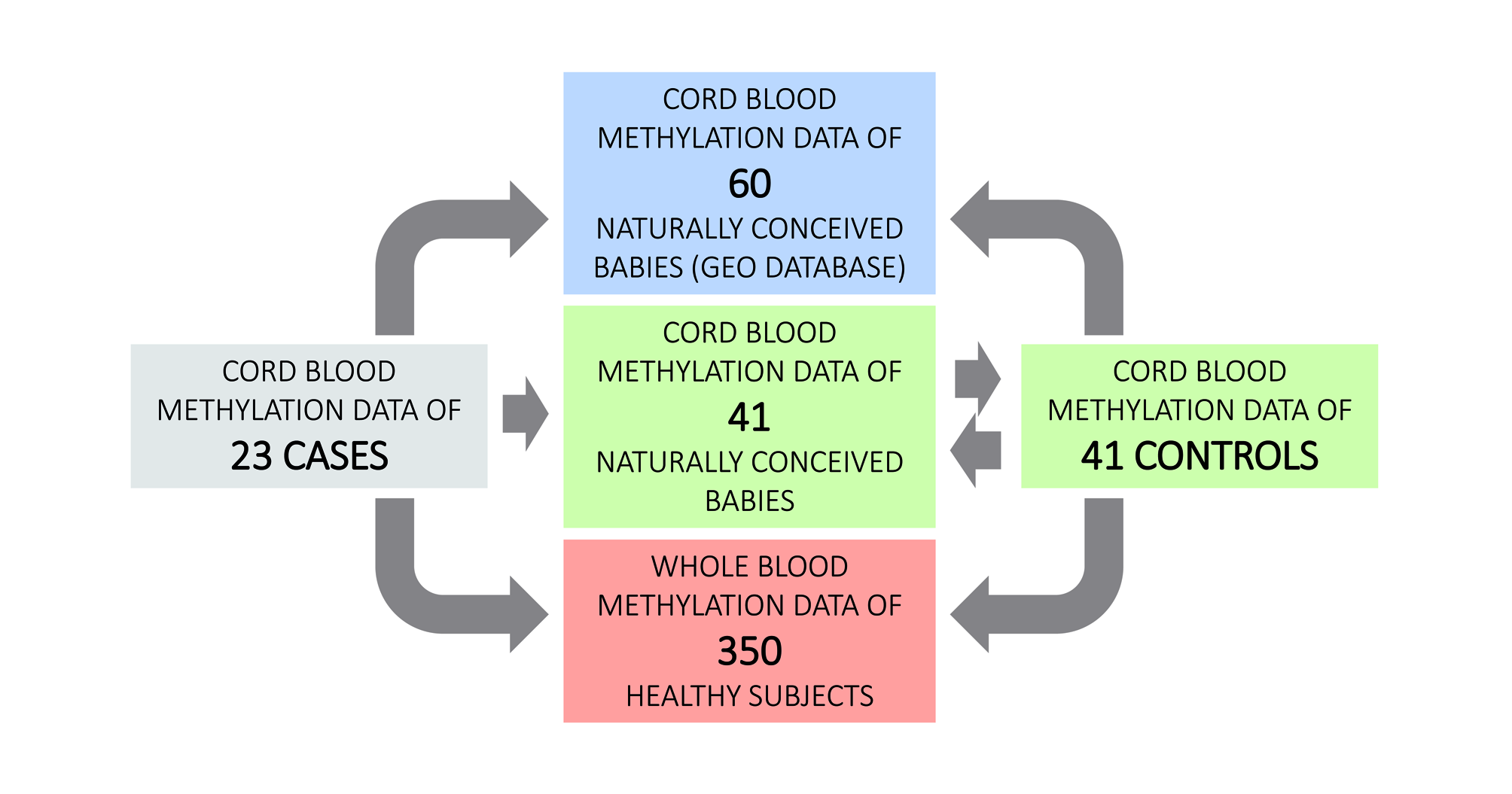

Supplement: Supplementary file 9 — Figure S8. Schematic description of the strategy used to estimate the number of SEVs. (TIF 982 kb) [file 13148_2018_510_MOESM9_ESM.tif]
